# Processing of Fresh-Cut Potato Using Plasma-Activated Water Prepared by Decreasing Discharge Frequency

**DOI:** 10.3390/foods12122285

**Published:** 2023-06-06

**Authors:** Aihemaitijiang Aihaiti, Ruxianguli Maimaitiyiming, Liang Wang, Jiayi Wang

**Affiliations:** Xinjiang Key Laboratory of Biological Resources and Genetic Engineering, College of Life Science and Technology, Xinjiang University, Urumqi 830046, China

**Keywords:** minimally processed produce, non-thermal processing, fresh-cut, browning, plasma-activated water

## Abstract

As a novel non-thermal processing method, the concentration of active compounds in plasma-activated water (PAW) is usually adjusted by changing the voltage and preparation time. We recently adjusted the discharge frequency and found that the PAW properties were improved. In this study, fresh-cut potato was selected as a model, and PAW was prepared using a frequency of 200 Hz (200 Hz-PAW). Its efficacy was compared with that of PAW prepared using 10 kHz. The results showed that the ozone, hydrogen peroxide, nitrate, and nitrite concentrations in 200 Hz-PAW were 5.00-, 3.62-, 8.05-, and 1.48-fold higher than those of 10 kHz-PAW. PAW inactivated the browning-related enzymes polyphenol oxidase and peroxidase, lowering the browning index and inhibiting browning; 200 Hz-PAW exhibited the lowest of these parameters during storage. In addition, PAW induced PAL to promote phenolic synthesis and increase antioxidant activity to delay malondialdehyde accumulation; 200 Hz-PAW exhibited the highest of these parameters. Moreover, 200 Hz-PAW had the lowest weight loss and electrolyte leakage rates. Furthermore, microbial analysis showed that the lowest aerobic mesophilic, mold, and yeast counts during storage were observed in the 200 Hz-PAW group. These results suggest that frequency-controlled PAW has the potential to treat fresh-cut produce.

## 1. Introduction

Consumers favor minimally processed produce (MPP) due to its convenience of use and different varieties. However, due to cutting, phenolic compounds flow out from the damaged cells and react with polyphenol oxidase (PPO) in the presence of oxygen to form quinone substances. This process, called browning, negatively affects the flavor and sensory color, leading to commercial value loss in MPP. To inhibit browning while maintaining the freshness of the produce, non-thermal technologies, including physical and chemical methods, have been applied. Among the physical methods, UV-C irradiation is commonly used. Wang et al. [1] reported that UV-C irradiation can inactivate PPO, peroxidase (POD), and phenylalanine ammonia-lyase (PAL) to inhibit browning in fresh-cut lotus root. Furthermore, the discoloration and browning rates in fresh-cut lettuce stems were reduced by UV-C irradiation, and this effect was attributed to PAL inactivation and not correlated with the POD and PPO activities [2]. Ascorbic acid (AsA) is an unchallenged low-cost and effective anti-browning agent for industry use. This agent can inhibit wound healing by modulating the activity of PAL, cinnamate-4-hydroxylase, 4-coumarate-CoA ligase, chalcone synthase, and coumarate-3-hydroxylase and by inhibiting browning. Another possible pathway was proposed by Zhao et al. [3]; they found that ATPase activities, ATP content, and energy charge were reduced after AsA treatment and that energy metabolism blocking was responsible for the inhibition of yam yellowing. The combined use of AsA with a physical method was recommended to increase the anti-browning effect. For example, a synergistic effect prevented browning in fresh-cut potatoes after treatment with vacuum packaging and AsA [4]. Furthermore, combining ultrasound and AsA more strongly inhibited POD and PPO activity in fresh-cut potatoes compared to single ultrasound or AsA treatment [5].

Plasma-activated water (PAW) is an emerging non-thermal technology prepared by treating water with a plasma jet and dielectric barrier discharge (DBD) plasma. Ar, He, and air can serve as the gas sources for plasma discharge; in particular, air is widely used due to its low cost and ease of use [6]. After using air in the discharge process at the gas–liquid interface, short-living species (OH^−^ and hydrated electrons) are formed in the water and subsequently react with each other to form stable ozone and hydrogen peroxide compounds [6]. Meanwhile, NO is formed at the gas–liquid interface and reacts with water to form the stable compounds nitrate and nitrite [7]. PAW has been used in MPP processing, and most studies have investigated microbial inactivation efficacy. *Escherichia coli*, present in spinach and kale, is reduced by approximately 6 log CUF/g after treatment with PAW prepared for 45 min [8]. The native microbes present on rocket leaves can be inactivated by 1.7–3 log CFU/g after washing with PAW for 2–5 min [9]. A synergistic effect was observed against *E. coli* O157:H7 after treatment with PAW plus propylparaben [10]. 

The browning-inhibitory effect of PAW has only recently been studied. For example, PPO and POD in fresh-cut apple slices are inactivated by PAW, inhibiting browning [11]. The concentration of sanitizing agents in PAW is generally associated with the discharge voltage, time, and plasma type [7]. Plasma is commonly generated under high discharge frequency [7]; however, our previous study (unpublished data) found that decreasing the discharge frequency using a pulsed wave generator improved the PAW properties. For example, the ozone, hydrogen peroxide, nitrate, and nitrite concentrations in PAW were highest after preparation under 200 Hz (approximately 5-, 3-, 8-, and 1.5-fold increases, respectively) compared with those of PAW prepared under 10 kHz. To our knowledge, the comparison of the browning-inhibitory effect of PAW prepared by decreasing the discharge frequency with that of high-frequency PAW has received little attention. We hypothesize that PAW prepared using this method will more strongly inhibit browning in MPP than PAW prepared using the high frequency (10 kHz) methods. In this work, fresh-cut potato was selected as a typical model, and the properties, including enzyme activity, quality parameters, and microbial counts, were analyzed to verify our hypothesis. 

## 2. Materials and Methods

### 2.1. Sample Preparation

The potatoes were purchased from a local market on the experiment day. The samples were rinsed and manually rubbed for 1 min under tap water to remove dirt. Then, the sample, skin-peeler, loading hopper, and cutting net were immersed in 200 ppm sodium hypochlorite (Sinopharm, Beijing, China) for 5 min. Sterilized water was used to rinse the samples and equipment to remove sodium hypochlorite residue. After peeling the samples and installing the loading hopper and cutting net, the sample was cut into cubes (1.5 × 1.7 × 0.8 cm) using an automatic cutter (Mond, Guangzhou, China).

### 2.2. PAW Generation and Sample Treatment

**Figure 1:**
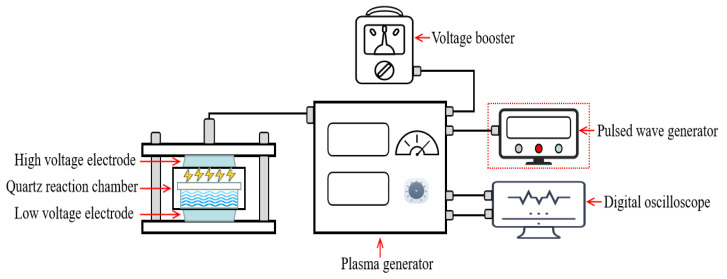
Schematic diagram of dielectric barrier discharge plasma system.

A DBD plasma system (Figure 1; CTP-2000 KP, Suman, Nanjing, China) was used to generate PAW. The high-voltage and low-voltage discharge electrodes were attached to the top and bottom of the quartz reaction chamber containing 50 mL distilled water, respectively. For the modified PAW preparation, the knob on the voltage booster was adjusted to set the input power (as calculated by the input current and voltage displayed on the plasma generator) to 50 ± 1 W. The pulsed frequency was adjusted to 200 Hz using a pulsed wave generator. The optimized discharge time was 10 min. The output voltage and current of the plasma generator were recorded using an oscilloscope, and the output power was calculated by integrating the obtained output voltage and current using Origin software (v. 2019; Origin Lab, Northampton, MA, USA). For high-frequency PAW preparation, the pulsed wave generator was removed, and the original discharge frequency (10 kHz) of the plasma generator was used. The preparation procedures were as described above.

After preparation, the PAW and potato cubes were immediately transferred into a sterilized centrifuge tube at a ratio of 5:1 (*v*/*w*) and processed for 2 min at 120 rpm on a vortex shaker. The sample was immersed in sterilized distilled water for 30 s to remove the PAW residue; the sample was dewatered in a sterilized manual salad spinner. Then, the sample was transferred into a polyethylene terephthalate plastic box and sealed using a polyvinyl chloride plastic film (Nanya, Taiwan, China) [12,13]. The sample was stored at 4 °C for 5 days for analysis [14].

### 2.3. Analysis of PAW Properties

The oxidation–reduction potential (ORP) and pH of the PAW were analyzed using a pH meter (PHS-3E; Inesa, Shanghai, China), while temperature and conductivity were analyzed using a conductivity analyzer (DDS-11A; Inesa, Shanghai, China). The ozone, nitrate, and nitrite concentrations were analyzed using test kits (Lohand, Hangzhou, China). For hydrogen peroxide analysis, the reaction mixture containing 0.1 mL PAW, 0.1 mL 10% titanium tetrachloride-hydrochloric acid solution, 0.2 mL concentrated ammonia water, and 0.9 mL distilled water was centrifugated at 12,000× *g* for 15 min at 4 °C, after a reaction for 5 min. The obtained precipitate was dissolved in 3 mL of 2 M sulfuric acid, and the absorbance at 412 nm was measured. 

### 2.4. Browning Index Analysis

The browning index analysis was performed according to the method reported by Perinban et al. [11]. Three samples were selected, and the L*, a*, and b* values for the six locations per sample were read using a colorimeter (CR400; Konica Minolta, Osaka, Japan). The browning index (BI) was calculated using the following formula:

$$BI=\frac{100\left(x-0.31\right)}{0.172}$$

where

$$x=\frac{a^{*}+1.75L^{*}}{5.645L^{*}+a^{*}-3.012b^{*}}$$

### 2.5. Weight Loss and Electrolyte Leakage Analysis

Electrolyte leakage was analyzed according to the method reported by Liu et al. [15], with minor modifications. The sample and the distilled water were transferred into a sterilized centrifuge tube at a ratio of 1:4 (*w*/*v*). After standing for 30 min, the conductivity value was measured (C1), followed by boiling for 20 min. The conductivity was measured again (C2) as the sample cooled to room temperature. Electrolyte leakage was calculated as C1/C2.

Weight loss was calculated as follows:

$$\mathrm{Weight}\;\mathrm{loss}\;\left(\%\right)=1-\frac{{\mathrm{Weight}}_{x}}{{\mathrm{Weight}}_{0}}$$

where Weight_*x*_ indicates the weight of the treated sample on the sampling day, and Weight_0_ indicates the weight of the treated sample on day 0. 

### 2.6. Liquid Nitrogen Grinding

On the sampling day, the fresh-cut potatoes were immediately immersed in liquid nitrogen for 30 s and transferred into an IKA analytical mill for further processing for another 30 s. The ground powder was used for the analyses in Section 2.7–Section 2.9.

### 2.7. PPO, POD, and PAL Analyses

The ground powder (5 g) was dissolved in 5 mL acetic acid-sodium acetate buffer (0.1 M, pH 5.5; 1 mM PEG, 1% PVP, 1% Triton X-100) and centrifugated at 12,000× *g* for 20 min at 4 °C. The PPO, POD, and PAL activities in the supernatant were analyzed as described by Liu et al. [15]. The enzyme activity was expressed as U/g on a fresh weight basis. 

### 2.8. Phenolic Content and Antioxidant Capacity Analyses

The ground powder (0.5 g) was dissolved in 5 mL 80% methanol and maintained for 30 min in the dark. After centrifugation at 12,000× *g* for 10 min, the phenolic content and antioxidant activity of the supernatant were determined as described by Wang et al. [16], and the results were defined as gallic acid equivalents (mg/100 g) and Trolox equivalents (μM/g) on a fresh weight basis, respectively.

### 2.9. Malondialdehyde Analysis

The ground powder (2.5 g) was dissolved in 5 mL trichloroacetic acid solution (10%) and centrifuged at 16,000× *g* for 20 min at 4 °C. The malondialdehyde (MDA) content in the supernatant was analyzed as described by Liu et al. [15], and the content was expressed as μM/100 g on a fresh weight basis.

### 2.10. Microbiological Analysis

The sample and the sterilized 0.85% NaCl were added into a stomacher bag at a ratio of 1:9 (*w*/*v*) and homogenized for 2 min to obtain a bacterial suspension. The diluted bacterial suspension (1 mL) was pour-plated into plate count agar (Hopebio, Qingdao, China) and incubated for 2 days at 37 °C to analyze the aerobic mesophilic count (AMC). For molds and yeasts (M&Y), 1 mL diluted suspension was pour-plated into rose bengal agar (Hopebio) and incubated for 5 days at 28 °C. The results were expressed as log CFU/g.

### 2.11. Statistical Analysis

The mean values among the different groups were analyzed using a one-way analysis of variance and the post hoc Duncan’s multiple range test in SPSS v.20. A *p* value of <0.05 was considered statistically significant. Each experiment was independently performed three times. A treatment with sterilized distilled water served as the control.

## 3. Results and Discussion

### 3.1. PAW Properties

When increasing the discharge frequency from 200 to 10 kHz, the output peak-to-peak voltage was decreased from 76.11 to 48.43 kV, and the peak-to-peak current was decreased from 0.08 to 0.03 A. Correspondingly, the output power was decreased from 28.30 to 13.21 W, leading to a higher temperature in 200 Hz-PAW (Table 1). The ozone and hydrogen peroxide concentrations of 200 Hz-PAW were 5.00- and 3.62-fold higher than those of 10 kHz-PAW, respectively; these increases were attributed to the higher ORP and conductivity value in 200 Hz-PAW. The nitrate and nitrite concentrations increased by 705% and 48%, respectively, as the frequency decreased from 10 kHz to 200 Hz, leading to a lower pH value in 200 Hz-PAW. This result shows that even if the input power is the same, when the discharge frequency is changed the output power will change, thereby changing the chemical and physical properties of the PAW. 

### 3.2. Effects on Color and Browning-Related Enzymes

**Figure 2:**
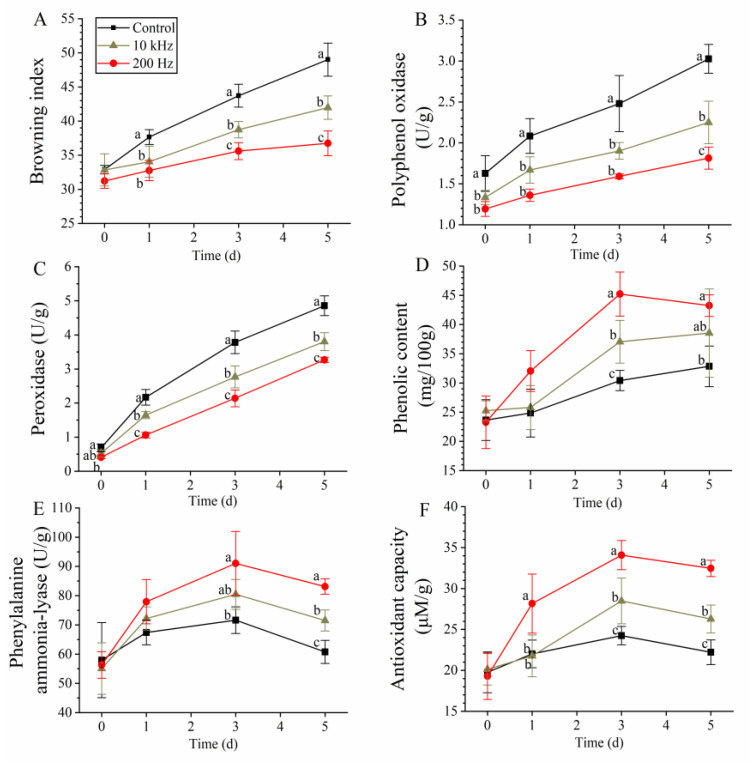
Browning degree, browning-related enzyme, and antioxidant properties in fresh-cut potato: (**A**) browning index; (**B**) polyphenol oxidase; (**C**) peroxidase; (**D**) phenolic content; (**E**) phenylalanine ammonia-lyase; (**F**) antioxidant capacity. Different lowercase letters within same day indicate significant differences (*p* < 0.05).

Color is the main factor determining whether consumers buy a product, and sensory browning is a major parameter used when evaluating the anti-browning effect. As shown in Figure S1, an appearance of browning was observed in the control at day 1, while a slighter appearance of browning was observed in the PAW-treated samples; at the end of storage (day 5), the control group lost its commercial value, while the 200 Hz-PAW appearance was more satisfactory than the control. Similar results were observed when further analyzing the color parameter using a colorimeter. The BI showed an increasing trend (Figure 2A), and the 200 Hz-PAW group had the lowest value from days 0 to 5. From days 3 to 5, the index in the 200 Hz-PAW group increased from 35.60 to 36.74, which was significantly lower than that of the 10 kHz-PAW group (which increased from 38.74 to 41.98). Beltrán et al. [17] evaluated the sensory color of fresh-cut potato treated with ozonated water and observed a lower score than that of the control. Another oxidizing agent, hydrogen peroxide, showed an anti-browning effect, reflecting its inhibitory effects on PPO and POD in fresh-cut produce [18,19]. AsA and citric acid also inhibited browning in fresh-cut produce [4,20]. PAW contains ozone, hydrogen peroxide, and acid; its anti-browning effect reflects a lower BI than that of the control [21], which is consistent with our results.

Resistance-related enzymes such as PPO and POD are responsible for the stress response in plants. PPO and POD can catalyze phenolic compounds in the presence of oxygen and hydrogen peroxide, respectively, to form quinone as a defense agent to fight against stress [22]. Thus, the browning extent was positively associated with the activity of PPO and POD, and wounding in fresh-cut produce induces the expression of PPO and POD during storage [23]. In this work, we also found that the activity of PPO and POD in all groups showed an increasing trend within 5 days (Figure 2B,C). 

PPO and POD are mainly active at the cut surface; acid and oxidizing agents can inactivate both [18,24]. As PAW is a solution containing an oxidizing agent and an acid, the PPO activity on day 0 was reduced by 18.40% and 26.99% in the 10 kHz-PAW and 200 Hz-PAW, respectively. From days 1 to 3, the PPO activity in the control increased from 2.08 to 2.48 U, while that in 10 kHz-PAW was 80.29% and 76.61% of the control and that in 200 Hz-PAW was 65.38% and 64.11% of the control, respectively. At the end of the storage, the PPO activity was lowest (1.81 U) in the 200 Hz-PAW group, which was significantly lower than that in the 10 kHz-PAW and control groups. Similarly, POD was significantly inactivated after treatment with 200 Hz-PAW, decreasing from 0.69 to 0.41 U (Figure 2C). From days 1 to 5, the lowest POD activity was observed in the 200 Hz-PAW group, and the activity on days 1 and 5 was only 48.85% and 67.28% of that of the control, respectively. 

Ozonated water inactivates PPO and POD activities in fresh-cut lettuce and celery, and the inactivation efficacy is associated with the ozone concentration [25,26]. A similar result was observed for hydrogen peroxide. Peng et al. [18] found that 0.9% hydrogen peroxide treatment leads to the lowest PPO and POD activities in fresh-cut Chinese water chestnut, compared with those in the sample treated with 0.15%, 0.3%, and 0.6% hydrogen peroxide. PPO was effectively inactivated by acid at pH values below 4.0 [27]. Calder et al. [24] found that an acid solution with a lower pH value accompanies higher PPO and POD inactivation activities. In this work, a lower pH value and higher ozone and hydrogen peroxide concentrations can explain the more effective inactivation activity of 200 Hz-PAW against PPO and POD compared to that of 10 kHz-PAW.

To increase the anti-browning effect, a combination strategy was applied. Physical methods are generally combined with a chemical agent or natural product. Recently, Xu et al. [5] observed an improved anti-browning effect after combining low-frequency ultrasound and AsA to process fresh-cut potatoes due to the greater inhibition of PPO and POD activities compared to that resulting from single ultrasound or AsA treatment. An enhanced inhibitory effect against PPO and POD activities was also observed after combining *Sonchus oleraceus* L. extract with ultrasound [28]. An interesting strategy using photodynamic treatments, including 420 nm light-emitting diodes and curcumin, was applied to fresh-cut potatoes, and the activities of PPO and POD decreased by 59.7% and 47.8%, respectively [29]. Although PAW was used as a single treatment, a significant inhibition of PPO and POD activities was observed without combination with other methods. Ozonated water at 2 ppm is ineffective in inactivating PPO in fresh-cut potatoes [24]. However, PAW containing 2 ppm ozonated water inactivates PPO activity by 32.3%; the treated sample had the lowest rate of increase during storage [11], indicating that other substances besides the ozone in the PAW contributed to the anti-browning effect, which was better than that which occurred when using single chemical agents. In this study, we modified the PAW preparation mode to increase the concentration of active substances, thereby enhancing the inhibitory effect on PPO and POD.

### 3.3. Effects on Antioxidant Capacity 

An increasing trend in the PAL activity was observed in all the groups from 0 to 3 days (Figure 2E). The 200 Hz-PAW group led the highest PAL activity from days 0 to 3, and the value on day 3 increased by 27.18% compared with that of the control. The phenolic content in the 200 Hz-PAW group increased by 21.94 mg GAE/100 g from days 0 to 3, whereas in the control group, it only increased by 6.78 mg GAE/100 g (Figure 2D). The antioxidant capacity in the control group increased from 19.76 to 24.24 μM/g in storage from 0 to 3 d, whereas that in the 10 kHz-PAW and 200 Hz-PAW groups was increased by 8.36 and 14.77 μM/g, respectively (Figure 2F). PAL is a key enzyme responsible for secondary metabolite synthesis (e.g., lignin and phenolic) on exposure to stress factors such as oxidative damage and wounding [30]. The antioxidant capacity was positively associated with phenolic content. Cutting can increase fresh produce phenolic content, PAL, and antioxidant activity during storage [15]. PAW, a solution containing reactive oxygen and nitrogen species (RONS), activates PAL to promote phenolic synthesis and correspondingly increase antioxidant capacity to scavenge ROS in fresh-cut produce [11]. Thus, the stronger stress (higher RONS concentration) caused by 200 Hz-PAW can explain the higher PAL activity, phenolic content, and antioxidant activity compared to those of the 10 kHz-PAW and control groups. 

### 3.4. MDA, Membrane Permeability, and Weight Loss

**Figure 3:**
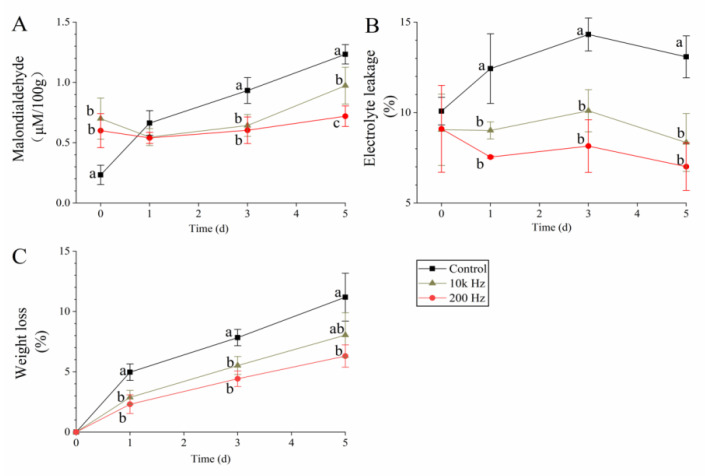
Membrane permeability, membrane oxidation extent, and weight loss in fresh-cut potato: (**A**) malondialdehyde concentration; (**B**) electrolyte leakage; (**C**) weight loss. Different lowercase letters within the same day indicate significant differences (*p* < 0.05).

Cuts and disinfectant treatment can cause cell damage on the cut surface, and MDA is an important indicator that reflects the extent of the oxidative damage of cell membranes [31]. The immediate MDA content increase and its lower values than those of the control during storage after PAW processing were observed by Perinban et al. [11]. Similar results were observed in this work; the MDA content immediately increased 2.61- to 3.04-fold after PAW treatment (Figure 3A). From days 0 to 1, the MDA content in the control group increased to 0.66 μM/100 g, which was similar to that in the PAW groups. In subsequent storage, the MDA content in all the groups increased, and the lowest increased rate was observed in the 200 Hz-PAW group. On day 5, the MDA content in the 200 Hz-PAW group was only 58.54% of that in the control group. Notably, antioxidant compounds are responsible for ROS scavenging; thus, the antioxidant level was negatively associated with the MDA content [32], which can explain the lower MDA level in the PAW groups (with a higher antioxidant capacity compared to control) compared to that of the control from days 1 to 5. 

The lower electrolyte leakage and weight loss observed by previous studies of fresh-cut potatoes during storage after anti-browning treatment [15,23] is attributed to washing-induced damage causing cell self-repair, tissue recovery, and electrolyte reabsorption [33,34]. Similarly, in this work, the lowest electrolyte leakage value was observed in the 200 Hz-PAW group from days 1 to 5, and the values in this group on days 1, 3, and 5 were 60.66%, 56.91%, and 53.59% of those in the control, respectively (Figure 3B). Weight loss in the 200 Hz-PAW group was the lowest on days 1–5, with an increase from 2.31% to 6.31%; in the control, an increase from 4.96% to 11.19% was observed (Figure 3C).

### 3.5. Effects on Microbial Counts

**Figure 4:**
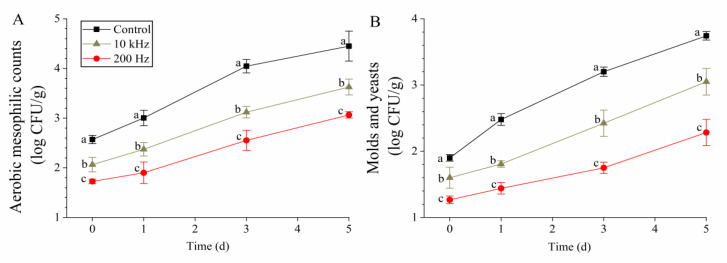
Microbial counts in fresh-cut potato: (**A**) aerobic mesophilic counts and (**B**) molds and yeasts. Different lowercase letters within the same day indicate significant differences (*p* < 0.05).

The AMCs on days 0, 1, 3, and 5 were reduced by 0.51, 0.63, 0.93, and 0.82 log CFU/g by 10 kHz-PAW and 0.84, 1.10, 1.49, and 1.38 log CFU/g by 200 Hz-PAW, respectively (Figure 4A). A similar trend can be observed in Figure 4B; 200 Hz-PAW led to an M&Y reduction of 0.63–1.46 log CFU/g from days 0 to 5, which was significantly higher than the count reduction achieved by 10 kHz-PAW. Oxidizing agents are sensitive to the organic load, leading to sanitizer overuse and ineffective disinfection of fresh-cut produce. For example, although browning inhibition was observed, Beltrán et al. [17] reported that ozonated water was ineffective in controlling the AMC and M&Y on fresh-cut potatoes. The disinfection of fresh-cut peppers using 3.5 ppm ozonated water reduced the AMC by 0.68 log CFU/g, whereas tap water led to a 0.66 log CUF/g reduction [35]. On a commercial scale, hydrogen peroxide is ineffective for fresh-cut produce washing, and metal ions (Cu^2+^, Ag^+^, and Zn^2+^) are needed to increase its efficacy [36]. However, PAW is an acid mixture containing oxidizing agents (ozone and hydrogen peroxide) and was effective for the AMC and M&Y control in this work; thus, it was consistent with previous studies [9,11]. 

## 4. Conclusions

In this study, a modified method (pulsed frequency control) was used to prepare 200 Hz-PAW, and its efficacy in processing fresh-cut potato was compared with that of 10 kHz-PAW. Both 200 Hz- and 10 kHz-PAW can inactivate PPO and POD to reduce the BI index and inhibit browning during storage (0–5 d). As a solution containing the highest RONS concentration, the lowest value for these parameters was observed in the 200 Hz-PAW group. The 200 Hz-PAW most strongly induced PAL to promote phenolic synthesis; correspondingly, it exhibited the strongest antioxidant activity to delay MDA accumulation. Moreover, 200 Hz-PAW led to the lowest weight loss and electrolyte leakage during storage. From days 0 to 5, the disinfection efficacy of 200 Hz-PAW was significantly higher than that of 10 kHz-PAW, achieving the highest count reduction of 0.84–1.38 log CFU/g and 0.63–1.46 log CFU/g for the AMC and M&Y, respectively. However, this work only analyzed the enzyme activity and BI index to reflect the anti-browning effect, and it lacked molecular analysis. To fully understand the browning-inhibition mechanism of PAW, transcriptomic and metabolomic analyses will be carried out in the future to provide further insights into the underlying molecular mechanism. It is a novel idea to use buffered water to prepare PAW instead of water. Future studies will employ the modified method to process buffered water, observe the changes in the active compounds, and use this solution to process fresh-cut produce.

## Figures and Tables

**Table 1:** Physical and chemical properties of PAW.

Parameter	Discharge Frequency (Hz)	
	10 k	200
pH	3.42 ± 0.19	2.64 ± 0.05
Temperature (°C)	23.80 ± 1.84	31.30 ± 0.75
ORP (mV)	461.67 ± 18.18	547.33 ± 9.02
Conductivity (μs/cm)	182.67 ± 16.80	883.33 ± 37.21
Ozone (mg/L)	1.21 ± 0.24	6.05 ± 0.73
Hydrogen peroxide (μM)	181.67 ± 40.41	658.33 ± 28.87
Nitrate (mg/L)	4.03 ± 0.19	32.45 ± 5.43
Nitrite (mg/L)	0.31 ± 0.04	0.46 ± 0.08

PAW, plasma-activated water; ORP, oxidation–reduction potential.

## Data Availability

The data presented in this study are available on request from the corresponding author (J.W.), upon reasonable request.

## Supplementary Materials

The following supporting information can be downloaded at: https://www.mdpi.com/article/10.3390/foods12122285/s1, Figure S1: Appearance of fresh-cut potato during storage.
