# Supplementary figures and images for: Processing of Fresh-Cut Potato Using Plasma-Activated Water Prepared by Decreasing Discharge Frequency
Source: Foods. 2023 Jun 6;12(12):2285. doi: 10.3390/foods12122285 (PMC10297012; doi:10.3390/foods12122285)

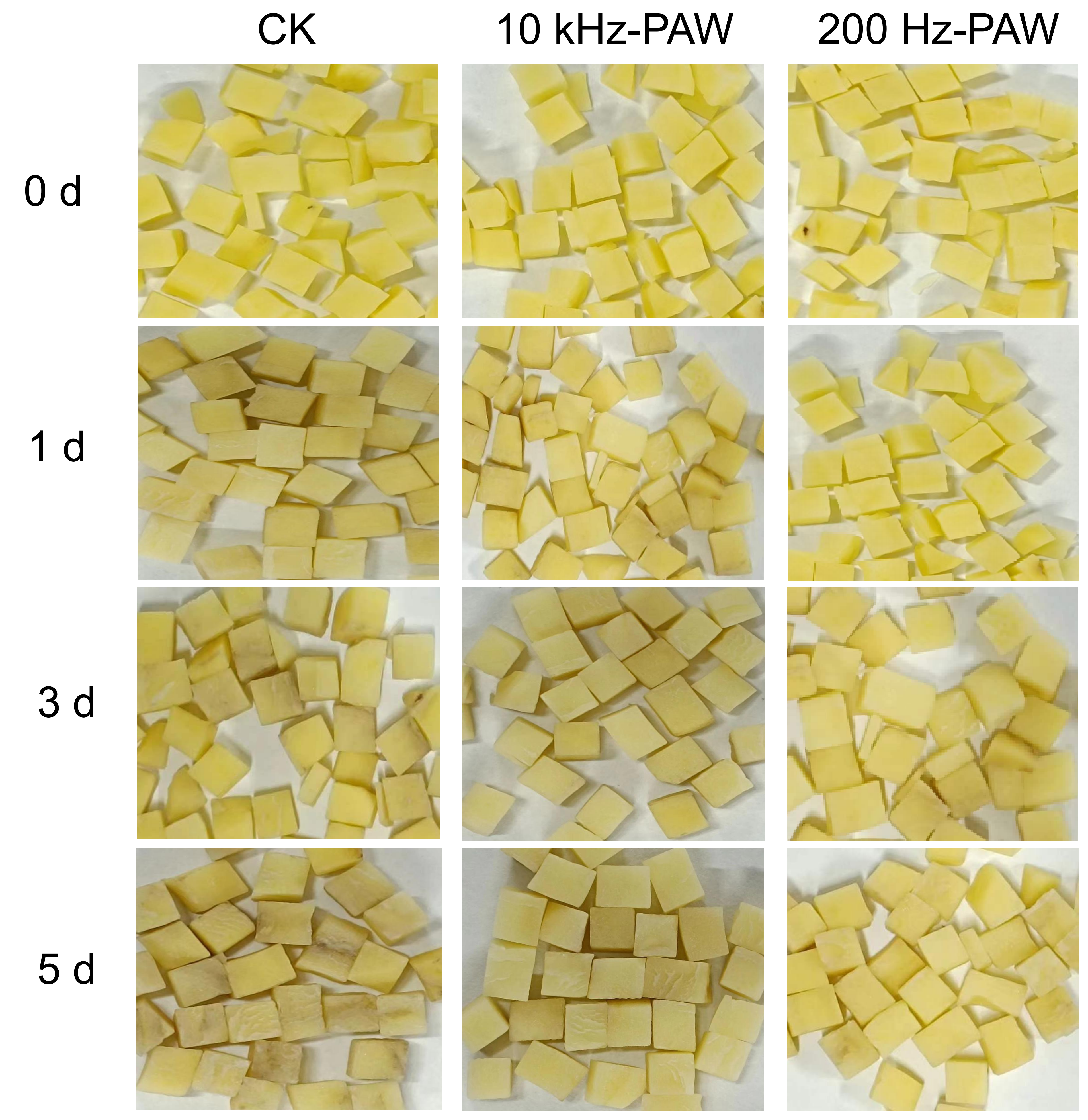

Supplement: Supplementary file 1 [file foods-12-02285-s001.zip › Figure S1.png]
